# Experiences of Mental Distress during COVID-19: Thematic Analysis of Discussion Forum Posts for Anxiety, Depression, and Obsessive-Compulsive Disorder

**DOI:** 10.1177/10541373211023951

**Published:** 2022-10

**Authors:** G. Brewer, L. Centifanti, J. Castro Caicedo, G. Huxley, C. Peddie, K. Stratton, M. Lyons

**Affiliations:** 1 Department of Psychology, University of Liverpool, Liverpool, United Kingdom

**Keywords:** anxiety, depression, COVID-19, mental distress, obsessive-compulsive disorder

## Abstract

The psychological impact of the COVID-19 pandemic on coronavirus patients, health care workers, and the general population is clear. Relatively few studies have, however, considered the impact of the pandemic on those with pre-existing mental health conditions. Therefore, the present study investigates the personal experiences of those with anxiety, depression, and obsessive-compulsive disorder during COVID-19. We conducted a qualitative study utilising Reddit discussion forum posts. We conducted three separate thematic analyses from 130 posts in subreddit forums aimed for people identifying with anxiety, depression, and obsessive-compulsive disorder. We identified a number of similar discussion forum themes (e.g., COVID-19 intensifying symptoms and a lack of social support), as well as themes that were unique to each forum type (e.g., hyperawareness and positive experiences during the pandemic). Findings should guide future practice and the support provided to those living with mental distress.

## Introduction

Researchers have documented the psychological impact of the COVID-19 pandemic on coronavirus patients and health care workers (Tan et al., 2020). An increase in mental distress is also apparent for the general population. For example, increased levels of anxiety, depression, self-harm, and suicide have been reported (Gunnell et al., 2020; Rajkumar, 2020; Sahoo et al., 2020). Indeed, the pandemic is likely to have impacted the general population in various ways, including social isolation, lack of usual community and professional support, financial stressors, and concerns over personal or loved ones’ well-being. The pandemic also has important consequences for those already experiencing mental distress (Shafran et al., 2020). Relatively few studies have, however, considered the impact of lockdown and COVID-19 on those with pre-existing mental health conditions.

Initial research indicates increased symptom severity amongst psychiatric patients during the pandemic and lockdown period (Hao et al., 2020). Further, those experiencing mental distress (e.g., anxiety, depression, obsessive-compulsive disorder) prior to the pandemic are more likely to have engaged in behaviour such as self-blame and are less likely to have employed adaptive strategies (e.g., acceptance) than non-patients (Rosa-Alcazar et al., 2021). It is important that the experiences of those with pre-existing mental distress inform mental health policy and practice (Zeilig et al., 2020). Therefore, in the present study, we consider the personal experiences of individuals who self-identify as having anxiety, depression, or obsessive-compulsive disorder during the COVID-19 pandemic utilising publicly available Reddit discussion forum posts.

### Anxiety and Depression

Quantitative studies show an increased prevalence of depression and anxiety during COVID-19 (Huang & Zhao, 2020; Nguyen et al., 2020). Research also demonstrates a worsening of symptoms in those with pre-existing mental distress (Fortang et al., 2021; González-Sanguino et al., 2020) and elevated COVID-19 related stress in psychiatric patients (Asmundson et al., 2020). A range of factors may contribute to worsening symptoms and stress in people who were depressed and/or anxious prior to the pandemic. For instance, quantitative studies demonstrate higher levels of depression and anxiety in people who feel lonely, have elevated COVID-19 worries, lack family support, have a loss of income, or have pre-existing health concerns with regards to the self or others (Liu et al., 2020; Palgi et al., 2020; Shevlin et al., 2020).

Frequent government briefings (typically reporting the number of people infected and deceased), widespread press coverage, and media commentary may also exacerbate anxiety (Dong & Zheng, 2020; Garfin et al., 2020). Indeed, research indicates that social media exposure during the pandemic is associated with elevated anxiety (Gao et al., 2020). However, it is unknown whether exposure to social media leads to increased anxiety or the other way around since individuals with anxiety seek out information about disasters (Weems et al., 2012). The relative impact of each stressor is also unclear. For example, Hamm et al. (2020) suggest that older adults with pre-existing depression were concerned about catching the virus, but social isolation was less of a concern (Hamm et al., 2020). Qualitative data is required to further inform our understanding of these issues and the personal experience of mental distress.

### Obsessive-Compulsive Disorder

The general fear of being infected could worsen the symptoms of individuals who possess features of obsessive-compulsive disorder. Indeed, for those with obsessive-compulsive disorder, being confronted with official guidelines may elevate health anxiety and disrupt attempts to control repetitive behaviour such as hand washing, cleaning, or touching objects (Abba-Aji, submitted; Banerjee, 2020; Davide et al., 2020). If people with obsessive-compulsive disorder find they are unable to source products such as hand sanitizers and disposable gloves (because of hoarding), this may also exacerbate distress. Initial research indicates a worsening of symptoms for many obsessive-compulsive disorder patients (Benatti et al., 2020; Jelinek et al., 2021; Tanir et al., 2020) and suggests that the COVID-19 pandemic may impact on the diagnosis and treatment of conditions such as obsessive-compulsive disorder (Fontenelle & Miguel, 2020). Further qualitative research is, however, required in order to understand the personal experience of obsessive-compulsive disorder at this time.

In the present study, we investigate the experiences of individuals who seek peer support in discussion forums during the COVID-19 pandemic. Having knowledge of personal experiences is crucial so that there is a shared strategy for helping the most vulnerable people. We expect this qualitative work to show the pandemic to have unique influences on the lives of people, depending on the type of difficulties they experienced before the outbreak.

## Methods

### Selection of Forum Posts

We utilised Reddit, a popular social networking discussion forum platform, which has more than 10,000 user-generated “subreddits”, i.e., online communities that are unified by common interests (Widman, 2020). The veil of anonymity and shared experiences make it easier for the users to openly talk about stigmatising issues that may be difficult to discuss face-to-face. Hence, Reddit has been successfully used to investigate sensitive topics such as eating disorder promotion (Sowles et al., 2018), incel communities (Maxwell et al., 2020), and mental distress (De Choudhury & De, 2014; Park et al., 2018). Further, previous research suggests that mental health related conversations on social media reflect actual crisis episodes experienced (Eichstaedt et al., 2018; Kolliakou et al., 2020; Reece et al., 2017).

We searched Reddit for subreddits related to mental distress by using the search terms “anxiety, depression, OCD”. We identified several relevant sites but chose those with the largest number of users for each type of distress (11,700 - 653,000 users at the point of data collection). We selected posts that were submitted between 1st March and 1st June, 2020. The sample sizes were 30 (anxiety), 50 (depression), and 50 (obsessive-compulsive disorder).

Upon entering each site, we searched for relevant posts using the terms “COVID, corona, virus, and pandemic”. After entering each search term, we went through the threads that discussed PERSONAL experiences of mental distress during the COVID-19 pandemic. We excluded posts focusing on the experiences of someone else, offering advice without sharing own experiences, or failing to mention COVID-19. We recorded posts by the usernames, analysing each username as one unit. The username, link to the post, type of distress (anxiety, depression, obsessive-compulsive disorder), age, sex, and country of origin of the posts (wherever this was possible) were recorded.

Using inductive thematic analysis (Braun & Clarke, 2006), researchers independently analysed the datafile. The researchers read the forum posts several times and established initial codes independent from each other. The researchers then discussed the codes, removed any duplicates, amalgamated similar codes, and investigated any discrepancies between the coders. After agreement on the coding system, we then organised the codes into broader themes in order to establish a preliminary thematic framework. It was clear that data saturation had been reached after analysis of the discussion forum posts collected.

### Ethical Issues

The subreddits we chose for the study were in the public domain, and the study did not require a review by the Institutional Review Board. However, when designing and conducting the study and reporting our findings we consulted relevant ethical guidelines, available guides to discussion forum research, and previously published discussion forum research (e.g., Smedley & Coulson, 2021). In particular, we considered the public nature of the information shared, the potential for benefit or harm, and the feasibility of seeking informed consent (Eysenbach & Till, 2001; Roberts, 2015).

We analysed posts available to the general public without registration or log in and adopted a number of measures in accordance with professional body guidelines (e.g., British Psychological Society, 2017) in order to protect the anonymity of the forum users. We are not revealing their online usernames, have slightly altered the wording of the quotes in this report, and do not reveal the names of the subreddits. We entered each quote into both Google (the most widely used search engine) and Reddit (the discussion forum platform used to obtain posts), and this did not lead to the original posts.

## Results

### Anxiety

#### Intense Fear of COVID

Those with anxiety often commented on an intense fear of COVID-19. This related not only to their own health but also to a fear that they would pass COVID-19 to loved ones. For example, one person commented “I’m convinced I’m going to get COVID and die, or my loved ones are going to die, and it’s taken over my life.” Similarly, another person stated:

I am scared to death that I have it and that I exposed them. I would feel even worse if they were to get sick or die from it, which has caused my anxiety to skyrocket and I’m having sporadic panic attacks.

Fears were exacerbated by sensitivity to symptoms and confusion between the symptoms of anxiety and COVID-19. For example, “Any changes in my body causes me worry.” and “Every time I feel something that could be a symptom it freaks me out. My anxiety makes it feel worse.” These COVID-specific fears were often prompted by media exposure. As one person described, “Is it just me or does the advert about the coronavirus give you anxiety. I'm not thinking about it until it appears on screen. I know it helps awareness about the virus but its triggering for me anyway”.

#### Fear of Returning to Normality

Those with anxiety typically commented that they had adapted well to lockdown but were anxious about the return to the ‘new normal’. The return to work was particularly stressful for some people. For example:

I have adapted really well to the social isolation and working from home … However, when I think about going back to the typical 8-5 grind with a 2-hour commute, I feel super anxious … I am just anxious at the thought of returning to the ‘normal life’ that I hated.

Similarly, another person posted “This morning they told us that tomorrow we will go back to the office. When I heard this the anxiety started growing fast  …  I just think about this and it scares me. I'm not ready to go back.” For others, the potential increase in social interactions caused anxiety. As described by one person:

It’s been a while since I’ve felt this good. But things in my city are slowly getting back to normal - just like my anxiety. I’ve been thinking about everything I will have to do again and I feel myself getting worse. I don’t really want this lockdown to end. Going outside my house everyday is such a huge challenge for me.

#### Isolation and Lack of Social Support

Although the thought of increased social interaction was stressful for some people, others found the lack of social support and isolation during the pandemic difficult. As described by one person:

I can feel people starting to get frustrated with me. They don’t know what to tell me now. It feels like there’s no reassurance or support anywhere, and I’ve ended up in a really dark place. Anytime I try to talk to anyone, I hear the same thing: ‘Everyone’s having a hard time right now.’

This issue was exacerbated when others were perceived to not take COVID-19 or anxiety seriously. For example:

My family tease and mock me for my fear of getting sick and dying from this disease. They’re also not super careful if they’re sick … Then because they didn’t respect my wish for space before they were noticeably ill, I get sick and it takes me months to recover … I’m tired of being teased for fearing sickness and the pandemic means my mental state is not good because everything makes me worry I’m sick. But I can’t say anything because I’m just being ‘silly’ or ‘a whiny baby’.

#### Overwhelmed and Exhausted

Forum posts often described a feeling of being overwhelmed, both by the pandemic and wider global events. Comments included:

This has been a horrible year. The virus, lockdown, riots, looting, and I just heard of some anti-LGBT bill the ‘president’ passed. I don't know what to make of anything.”, and “I see bad news everywhere. There’s no break. There’s another number, another case, another death. I’m tired of it and it’s all out of my control.

As a consequence, people often reported feeling exhausted and a sense of desperation or hopelessness. For example, “I’m so exhausted. The extreme uncertainty is more worrying than the worst possible outcomes” and “I'm so sick of every decision carrying so much weight. Going to the store, ordering food, pressing a button on an ATM. This is so exhausting.” As summarized by one person, “I cry a lot at the moment. I feel hopeless and I can't get out of this grey and black vision of the future.”

### Depression

#### Positive Experiences During the Pandemic

Many forum posts described positive experiences, particularly associated with the lockdown. This often reflected distance from specific stressors such as work or routine social interaction. As described in one forum post:

I don’t have to go out and pretend to be a happy social person, no pretending to care about people’s lives, fake smiles and awkward small talk. Before, I constantly felt so drained … . In quarantine I’m not drained by anyone, I’m not required to do anything except my own work. I’ve always dreamt of having a place in the woods and being a total recluse or being a monk in the mountains somewhere and having zero bullshit. Quarantine has zero bullshit. I love it, people don’t talk out of risk for their health, I wish I’d been quarantined the last 5 years.

Although the benefits of lockdown were recognized, some people were also concerned about the lifting of lockdown and the return to ‘normal’. For example:

Lockdown has been a way for me to escape from everything. It’s like the whole world is put on pause for me. For me to heal emotionally … Now that it’s being lifted I … have no idea what to do. It doesn’t help that everyone’s been so excited and ready to meet their partners and friends. And me … I feel more alone than normal.

Another person explained “I feel as if everyone’s excited and making plans for when lockdown ends and I’m here with my stomach twisting dreading every mention of street parties, gatherings etc etc. I’m not ready for my normality to start being abnormal again.”

#### Intensifying Depression Due to COVID

Posts frequently commented on the experience of increased depression and mental distress. For example, “My depression has rocketed … I've been in tears for weeks. I've had depression for years but I feel like this has escalated it even more” and “The depression is out of control. I keep sitting in my room waiting for the end of the world to come.” Increased distress reflected both the pandemic itself and associated events such as unemployment. As described in one post:

This virus has destroyed my business. I’m hardly making ends meet. I’ve been alone in my home for two months. I feel like I don’t have a purpose. I don’t even want to get out of bed anymore. I feel pathetic and a failure.

Another person commented, “Lost my job recently due to the economy crapping itself. Having nothing to do has really sent me into a downward spiral … I don't feel like I exist anymore”. As a consequence, people often demonstrated catastrophizing, reporting an overwhelmingly negative view of the world, with everything feeling ‘pointless’. For example:

I have no career, I have no future, I have no joy anymore. I lost my job, my relationship, the home I built for myself … I feel no love or hope anymore. and “Fuck this shit. I hope the whole world burns.

#### Isolation and Lack of Social Support

A common theme was the sense of isolation and lack of social support experienced by those with depression. For some, this involved a separation from their social support network. For example, “Being separated from my work family has been really hard”. For others, there was a sense of rejection: “Everyone in my house says I give a bad vibe and that they don’t want to be around me. I don’t blame them because it’s draining being around me.” Another person commented:

No-one messages me first or cares about me. If I do open up then it makes it worse. I told people on Thursday night that I was feeling suicidal and got a quick ‘take care of yourself’ and nothing since. For all they know I could be dead and it's clearly not important. I've been hit hard because I had made what I thought was a reliable group of people who I really thought 'got it' but it feels that everyone is so caught up in their own stuff that I've been forgotten. When they know about my mental health issues and that I'm alone during lockdown, how hard is it to just check in every couple of days?

Conflict was also evident. As explained by some people, “I need to stay at home with my parents 24 hours a day 7 days a week and sometimes it's suffocating. Especially when I argue with them but still have to act happy.” and “I really hope this ends quickly so I can get the fuck out of this toxic house.”

#### Disrupted Coping Strategies Due to COVID

In addition to the lack of social support, the pandemic disrupted other coping strategies such as exercise. As described by one person:

Working out at the gym had helped the depression and my confidence was beginning to grow. With the pandemic and the lockdown my depression and anxiety are coming back. Being unable to go to the gym for the last few months really did affect me.

Access to external support and therapy also became problematic. For example “I’ve not been able to visit my GP to tell them what happened … and not been able to get a psychiatric referral because I can’t get the GP appointment and because psychiatrists aren’t working one-to-one because of the lockdown.” Another person reported, “My therapist got COVID and I haven’t been able to see them in a couple of months. We were going to change my medication once my next appointment was scheduled so I haven’t been taking any.”

### Obsessive-Compulsive Disorder

#### Increasing Obsessive Behaviour Due to COVID

The most commonly reported issue discussed in these online forums was the impact of COVID-19 on obsessive behaviour. This was typically centered on the fear of contamination and ritualized cleaning or handwashing although other non-hygiene related obsessive behaviour also increased. As stated in one post:

The last week COVID has made me worse again. I feel I need to wash my hands or sanitise everything after I touch things, and I'm only in my house. It's driving me crazy, I'm obviously wiping surfaces, taps, door handles etc down regularly but I still feel as if I need to wash and feel clean otherwise I can't relax.

Another commented:

Once the coronavirus started my OCD has been at a record high. I clean everything in the apartment absolutely every day … all night all I could think about is how today I need to go to the shops (which I don’t want to do) and get more cleaning supplies and clean everything again today. I don’t want to give into the compulsions, but when COVID is around all I can think is ‘they could have the virus and not know it” … I hate living like this, I’m so miserable.

The intensification of symptoms was such that people “Worried it will be years even after the pandemic goes away before I get back to controlling my OCD again.” Forum posts noted that obsessive-compulsive disorder symptoms had been under control prior to the pandemic; there was frustration at the worsening of symptoms. For example, “It's been quiet for so long, now it feels as if I’m back at square one” and:

I have worked so fucking hard to lower my handwashing in the past year, and I'm getting so frustrated with myself for giving into it more and more recently. I've gone back to aggressive scrubbing all the way up my forearms when I wash, I'm thinking more obsessively about how I'm ‘contaminated’, and I'm exhausted from it.

#### COVID Influencing Anxiety and Distress

Unsurprisingly, forum posts often described the intensification of anxiety and distress during the pandemic. For example, “I'm dealing with nearly constant heightened anxiety” and “I feel scared and really overwhelmed by everything. It all feels so heavy.” Although their own risk of contamination was discussed, people were also extremely anxious about the safety of their loved ones. As summarised in one post:

I just can't stop thinking that everyone I love is about to die. I live away from my family and I keep thinking that I'll never see them again. I can't stop imagining my partner getting COVID, getting sick and dying. I keep imagining myself dying in the hospital alone. I can't imagine reaching the end and not having someone there or being able to hold a loved one's hand. I'm so scared of loss and death and don't know how I would cope with losing a family member or my partner.

Another stated:

I can’t stop thinking that this is the end. I am really anxious that they have caught the virus and will die, and that I won’t be able to see them or say goodbye to them before it happens. I can’t sleep. I’ve had multiple panic attacks … I have been calling them non-stop to check on them and I’m constantly questioning them to see how safe they’re being.

#### Normality and Justification

For some people, the extra safety steps taken by the general population (handwashing, mask wearing etc.) reassured them that their behaviour was normal and their fears justified. For example, “Being advised to do all the things that I did anyway almost gives me a sense of normality. I haven’t felt normal in years.” and “It’s funny how the same people that made fun of me for not touching doorknobs, only eating off paper or plastic plates and silverware, and washing my hands till they bleed are becoming more and more ‘like me’.” For some people, however, there was a difficulty knowing when their behaviour was appropriate and when it became dysfunctional. As summarized by one person:

Being more conscious about germs and washing hands more regularly isn't necessarily a bad thing at the moment. Arguably we are positioned better than most of the population in terms of protecting ourselves because we already are thinking so much about germs and contamination. But I'm at that point where my increased caution is no longer useful and has begun to hinder my daily functioning again.

#### Hyperawareness

An increased awareness of bodily functions (e.g., breathing) was apparent, with people often finding it difficult to determine if physical sensations were an indication of COVID-19. For example:

During the pandemic I became hyper aware of how I was breathing because of a shortness of breath being one of the symptoms. I found myself always checking to make sure it was okay and generally it was. I realised I couldn't stop thinking about my breathing … It's been going on for about a month and has been manageable some days, almost non-existent and some days totally debilitating as though if I stop these thoughts I'll just stop breathing.

Similarly, another person explained:

I have got asthma and whenever it’s difficult for me to breathe I’m convinced that I have COVID. I get cramps because of other health conditions and I convince myself I have COVID. Other pre-existing health conditions are also making me freak out.

This could lead to obsessive health checking such as “checking my temperature about ten times a day” and “compulsively scheduling visits with the doctor”.

#### Interpersonal Conflict

Those with obsessive-compulsive disorder frequently described conflict with family, partners, or roommates. Conflict was typically associated with cleaning rituals or others being perceived to not take the risk of COVID-19 contamination seriously. One person commented:

During the pandemic I've noticed more of my family's behaviour, particularly when they don't wash their hands after doing certain things. It's gotten to the point that if I ask them to wash their hands, I get shouted at. For example, my sister will take her hands and rub them on doorknobs or the stairway rails … I had a bad episode today that resulted in my family not speaking to me for the night.

Another explained:

All I can think about is how they are contaminating everything and they have germs on them and I need to clean everything … I ended up shouting at my roommate and told him not to have people over here and I spend all my time cleaning and he doesn’t. I apologized to him later but I do feel bad for talking to him like that, I just couldn’t cope with it and I took all my OCD stress out on him.

The pandemic was particularly distressing for those with families who believed COVID-19-related conspiracies. For example:

At first everyone was fine and wore a mask and stuff … for a couple days. Then my family jumps on the conspiracy bandwagon and thinks that the virus is a hoax, or it’s because of 5G, or something to do with vaccines, or a combination or all of those. I’ve tried to explain to my parents that the virus is real (they sometimes believe that but their argument changes daily) and that we should continue to wear a mask. But they refuse and say that they're not going to follow what the government says and they think that this virus is a plot to take their freedom away.

## Discussion

The present study investigated the lived experience of three mental health conditions (anxiety, depression, and obsessive-compulsive disorder) during the COVID-19 pandemic. We analysed themes for online forum posts for each condition separately. For anxiety we identified fear of COVID, fear of returning to normality, isolation and lack of social support, and feeling overwhelmed and exhausted. For depression, the themes were positive experiences during the pandemic, intensifying depression due to COVID, isolation and lack of support, and disrupted coping strategies due to COVID. Posts relating to obsessive-compulsive disorder referred to increasing obsessive behaviour due to COVID, anxiety and distress, normality and justification, hyperawareness, and interpersonal conflict.

There were important similarities across conditions for the period in which we examined the online posts. In particular, COVID-19 impacted on the intensity or type of mental distress experienced for all groups although the nature differed across conditions. For those with anxiety, there was a heightened fear of COVID-19 and also a fear of returning to ‘normality’ and social interaction; anxieties around returning to normality were also true for depression. Those with depression reported an intensification of symptoms related to a negative view of the world and catastrophizing and less about fear, whereas posts related to obsessive-compulsive disorder described the extent to which COVID-19 had impacted on the fear of contamination and obsessive cleaning or hand washing. It is important, therefore, for those providing support to those living with mental distress to acknowledge the extent to which conditions may be shaped or influenced by COVID-19.

Another shared experience was the impact of COVID-19 on personal relationships and the availability of social support. Conflict with those who were perceived to not take the pandemic seriously was reported by those with anxiety and obsessive-compulsive disorder. A feeling that loved ones had ‘had enough’ and were no longer willing or able to provide support was evident in the anxiety and depression posts. Therefore, it is important for those supporting patients or loved ones with mental distress to acknowledge the impact of COVID-19 on access and need for social support.

### Limitations and Future Research

The present study relied on information posted on online discussion forums. It is important to note that there is high comorbidity of mental health conditions; it is likely that people posting in one forum experienced more than one type of distress (e.g., Fenton, 2001). Similarly, although many people explicitly mentioned contact with medical professionals or therapists, we do not have confirmed diagnoses or medical histories for those posting in the online forums. Regardless of formal diagnosis, our findings suggest that people utilise online resources such as discussion forums to obtain support. Thus, future research should investigate opportunities to use telemedicine during pandemics (Torous et al., 2020; Zhou et al., 2020), particularly when targeted at vulnerable groups.

To conclude, the COVID-19 pandemic poses significant challenges to individuals with pre-existing mental health conditions. Although there were similarities in COVID-19 experiences (e.g., exacerbation of the symptoms) across different types of distress, some of the difficulties were specific to the type of distress the individual experienced. Perhaps most importantly, the threat posed by COVID-19 remains, and even in countries where infection rates or mortality appear to have peaked, there is a risk of further outbreaks. It is important to assess the lived experience of those with mental distress during the pandemic. This knowledge can inform clinical practice as lockdown measures are removed (increasing access to therapy) and to support people in the event of future outbreaks.
